# *Solanum paranense* Extracts and Solanine Present Anti-Inflammatory Activity in an Acute Skin Inflammation Model in Mice

**DOI:** 10.1155/2017/4295680

**Published:** 2017-05-24

**Authors:** Mariana Piana, Camila Camponogara, Aline Augusti Boligon, Sara Marchesan Oliveira

**Affiliations:** ^1^Laboratório de Análises Fitoquímicas, Programa de Pós-Graduação em Ciências Farmacêuticas, Universidade Federal de Santa Maria, 97.105-900 Santa Maria, RS, Brazil; ^2^Laboratório de Neurotoxicidade e Psicofarmacologia, Programa de Pós-Graduação em Ciências Biológicas: Bioquímica Toxicológica, Universidade Federal de Santa Maria, 97.105-900 Santa Maria, RS, Brazil

## Abstract

The aim of the study was to evaluate the anti-inflammatory activity of the* S. paranense* crude extract,* S. paranense* alkaloid fraction, and solanine alkaloid. These samples reduce the croton oil-induced ear edema in a dose-dependent manner and a maximum inhibition of 81%, 98%, and 80% in the doses of 1.0, 0.73, and 0.37 mg/ear, respectively. Moreover, the samples inhibit the MPO activity with an inhibition maximum of 51%, 40%, and 46% in the doses of 1.0, 0.73, and 0.37 mg/ear, respectively. Similar results were found for dexamethasone 0.10 mg/ear (positive control), which showed inhibitions of ear edema and MPO activity of 100% and 65%, respectively. These results found probably are related to the presence of solanine which is present in significant quantity in the alkaloid fraction and others as rutin and rosmarinic, chlorogenic, and gallic acids. These results support the use of* S. paranense* for the treatment of inflammatory skin disorders.

## 1. Introduction

The skin is the first interface between the body and the environment, providing the primary defense against microbial pathogens and injuries through physical, biochemical, and immunological mechanisms [1]. The regulation of these mechanisms is essential because they are implicated in the pathogenesis of several topical inflammatory disorders [2].

In the inflammatory process, the high levels of inflammatory cytokines and reactive oxygen species are produced and maintained by an interaction of various inflammatory cells that migrates to the inflammation site in response to the release of proinflammatory mediators [2]. This way, the extracts represent potential for the treatment of topical inflammatory diseases because substances from plants, such as phenolic acids, alkaloids, flavonoids, terpenes, and catechins, are known to modulate the expression of proinflammatory signals due to the capacity to inhibiting enzymes and proinflammatory mediators [3].

Several studies showed that* Solanum* species has anti-inflammatory activity, like* Solanum corymbiflorum* [4] in an acute skin inflammation model,* Solanum lycopersicum* [5] and* Solanum trilobatum* [6] in a carrageenan-induced paw edema rat model, and* Solanum nigrum* [7] in a subacute granuloma inflammation model. However, many species of this genus have no research about its anti-inflammatory activity.* Solanum paranense *Dusén is among such species, popularly known as Joá-velame, belonging to genus* Solanum* (family* Solanum*) [8], and until the moment, there are no studies related to this species. Taking into consideration these facts, the aim of this study was to evaluate the topical anti-inflammatory activity of the* S. paranense *crude extract,* S. paranense *alkaloid fraction, and the solanine alkaloid on an acute model of skin inflammation in mice and relate this activity with its constituent compounds.

## 2. Material and Methods

### 2.1. Drugs

The following drugs were used to execute the experimental protocols: Croton oil, hexadecyltrimethylammonium bromide (HTAB), tetramethylbenzidine (TMB), dexamethasone, rutin, gallic acid, ascorbic acid, chlorogenic acid, rosmarinic acid, solanine, DPPH, ammonium phosphate monobasic (all from Sigma, St. Louis, MO, USA), hematoxylin-eosin, acetonitrile, butanol, paraffin, ethanol, and Folin-Ciocalteu (all from Merck, Whitehouse Station, New Jersey, USA), Isoflurane (Baxter, São Paulo, Brazil), sodium acetate, acetone, acetic acid, and formaldehyde (all from Vetec, Rio de Janeiro, Brazil).

### 2.2. Plant Collection and Extractions

Leaves of* S. paranense* were collected in Gaurama (Rio Grande do Sul State of Brazil) in February (2013). A dried voucher specimen is preserved in the herbarium of the Department of Biology at Federal University of Santa Maria (register number, SMBD 13748). The leaves (500 g) were dried and powdered in a knife mill; this material was macerated at room temperature with 70% ethanol for a week, with daily shake-up. After filtration, the hydroalcoholic extract was evaporated under reduced pressure to remove the ethanol; this was taken to complete dryness in stove (temperature below 40°C), yielding the extract.

The quantity of 1.50 g of extract was used to get the alkaloid fraction, according to the method described by Sotelo and Serrano [9], with modifications.

### 2.3. Phytochemical Compounds

#### 2.3.1. HPLC Analysis of Phenolic Compounds on* S. paranense* Extract

The phenolic compounds analysis was performed by high performance liquid chromatography (HPLC), through the method described by Zadra et al. [10], slightly modified in a Shimadzu Prominence system (Kyoto, Japan) equipped with a SIL-20A autosampler, equipped with Shimadzu LC-20 at reciprocating pumps connected to the degasser DGU 20A5 with integrator CBM 20A, UV–VIS detector DAD SPD-M20A, and Software LC Solution 1.22 SP1. Reverse phase chromatographic analyses were carried out under gradient conditions, using a C-18 column (4.6 mm × 250 mm) packed with 5 mm diameter particles; the mobile phase 1 was acetic acid 2.0% in water and mobile phase 2 was methanol. All solutions, mobile phase, and samples were firstly dissolved in the mobile phase and filtered through a 0.45 mm membrane filter (Millipore). The chromatographic peaks were confirmed by comparing their retention time and Diode-Array-UV spectra with those of the reference standards chlorogenic acid (327 nm), gallic acid (272 nm), rosmarinic acid (330 nm), and rutin (355 nm). The flow rate was 0.6 mL/min, and the injection volume was 40 *μ*l. All chromatographic operations were carried out at room temperature and in triplicate.

#### 2.3.2. HPLC Analysis of Solanine on* S. paranense* Alkaloid Fraction

HPLC analysis of solanine was carried out according to Sotelo and Serrano [9] in the same equipment described previously. Reverse phase chromatographic analysis was carried out under isocratic conditions, using a C-18 column (4.6 mm × 150 mm) packed with 5 mm diameter particles. The mobile phase was acetonitrile -0.05 M monobasic ammonium phosphate buffer (3 : 7, v/v), at pH 6.5. The solvent flow was 1.2 mL/min and an injection volume 20 *μ*L. The quantification was performed using the method of the internal standard. The chromatographic peaks were confirmed by comparing their retention time and Diode-Array-UV spectra with reference standard solanine at 200 nm. All chromatographic operations were carried out at room temperature and in triplicate.

### 2.4. Animals

Male adult Swiss mice (25–30 g) were used in the experiments. The animals were provided by the Central Biotery of the Federal University of Santa Maria. After the acquisition, they were divided into groups and kept in a temperature-controlled room (22 ± 2°C) under a 12 h light-dark cycle. Animals were acclimatized to the laboratory for at least 1 h before the experiments and were used only once. All of the experiments were carried out between 8:00 a.m. and 5:00 p.m. The data reported in this study were carried out in accordance with national and international legislation (guidelines of Brazilian Council of Animal Experimentation, CONCEA, and U.S. Public Health Service's Policy on Humane Care and Use of Laboratory Animals, PHS Policy) and with the approval of the Ethics Committee for Animal Research of the Federal University of Santa Maria (process 6481091215/2016). The animals were separated into 6 groups in each experiment with 6 animals by the group, totaling a final number of 150 animals. The number of animals and the amount of irritant agent were the minimum necessary to demonstrate the consistent effects of the drug treatments.

### 2.5. Inflammatory Parameters Measurements

Skin inflammation was induced by topical application of croton oil, and the inflammatory response was assessed through edema formation, infiltration of inflammatory cells (myeloperoxidase activity), and histological procedure.

### 2.6. Treatments

The* S. paranense* crude extract (0.01–1 mg/ear),* S. paranense* alkaloid fraction (0.01–0.73 mg/ear), solanine (0.001–0.37 mg/ear), or dexamethasone (0.1 mg/ear; used as a positive control) was dissolved in 20 *μ*L of acetone and applied topically before the croton oil treatment. The animals ear thickness was measured before and after the application of the irritant agent [4]. Six hours after the treatment with croton oil, the animals were sacrificed, and ear samples (circles of tissue 6 mm in diameter) were collected for further analysis.

### 2.7. Croton Oil-Induced Ear Edema

The ear edema was induced by a unique topical application of croton oil at a concentration of 1 mg/ear in the right ear of the mice and was manifested as an increase of ear thickness [4]. The* S. paranense* crude extract, alkaloid fraction, solanine, or dexamethasone was applied topically immediately before the croton oil treatment. The thickness was measured before and after induction of the inflammatory response using a digital micrometer (Digimess) in animals anesthetized with isoflurane [11]. The micrometer was applied near the tip of the ear just distal to the cartilaginous ridges. The thickness was represented by the variation before and 6 h after treatment and expressed in *μ*m. To minimize variation, a single investigator performed the measurements throughout each experiment.

### 2.8. Myeloperoxidase Activity (MPO) Assay

The myeloperoxidase activity is used as a biochemical marker of polymorphonuclear leukocyte influx (mainly neutrophil) to the injured tissue. MPO activity was determined using an assay described previously [11, 12]. After six hours of application of croton oil, the MPO enzyme activity was assessed in the ear samples. Tissue samples were homogenized with a motor-driven homogenizer in acetate buffer (8 mM, pH 5.4) containing HTAB. For evaluation of MPO activity, the supernatant was incubated with TMB (18.4 mM) at 37°C for 3 min. The enzyme activity value was assessed colorimetrically at 630 nm using a microplate reader (Fisher Biotech BT, 2000). The results were expressed as optical density (OD)/mL of the sample.

### 2.9. Histological Assessment of Skin Tissue

To verify the histological changes in the mice ear after irritant agent and treatments application, samples were collected six hours after the induction of inflammation. Mice were euthanized, and ear tissue was removed and fixed in alfac solution (a 16 : 2 : 1 mixture of ethanol 80%, formaldehyde 40%, and acetic acid). The ears were subsequently embedded in paraffin, sectioned to 5 mm, and stained with hematoxylin-eosin. The leukocytes infiltration was assessed in representative areas selected with 20x and 40x increments. The quantification of leukocytes in the tissue (dermis) was performed by counting the cells per field, and five fields from three distinct histological sections of each group were analyzed. To minimize a source of bias, the investigator analyzed the specimens blindly [4].

### 2.10. Statistical Analysis

The results are presented as mean ± SEM with exception of the ID_50_ values (dose required to reduce the responses of the treated groups by 50% relative to the control group), which are reported as geometric means plus their respective 95% confidence limits. The maximum inhibitory effect (*E*_max_) was calculated based on the response of the control groups. The statistical significance between the groups was assessed by one-way analysis of variance (ANOVA) followed by a post hoc Newman-Keuls test. For phenolic composition calibration curves were used; the experimental values were expressed as mean ± SEM (*n* = 3). For solanine mean ± SEM (*n* = 3) was used. All tests were carried out using GraphPad 5.0 Software (San Diego, CA, USA). The accepted level of significance for the test was *P* < 0.05.

## 3. Results

### 3.1. Phytochemical Analysis

**Figure 1:**
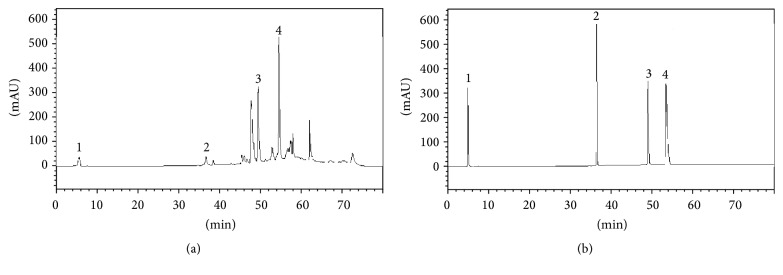
Chromatogram* S. paranense* extract (a) and the chromatogram of standards (b). 1 corresponds to gallic acid peak, 2 chlorogenic acid, 3 rosmarinic acid, and 4 rutin.

The yield of the extract was 8.83% (w/w). In the analysis by HPLC, it was possible to quantify gallic acid (6.41 ± 0.15 mg/g), chlorogenic acid (5.74 ± 1.00 mg/g), rosmarinic acid (9.43 ± 0.29 mg/g), and rutin (21.85 ± 0.45 mg/g) (Figure 1).

**Figure 2:**
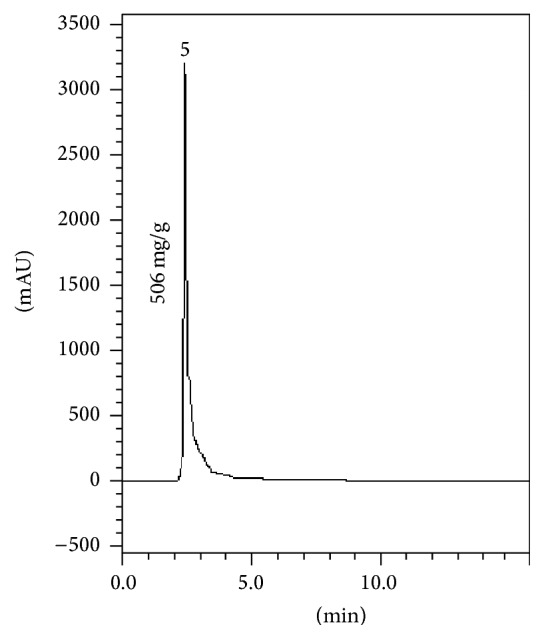
Chromatogram of* S. paranense* alkaloid fraction. 5 corresponds to solanine.

The alkaloid fraction showed a yield of 73% (w/w) in relation to the extract and analysis by HPLC 506 ± 1.75 mg/g of solanine in the* S. paranense* alkaloid fraction and consequently 371.06 mg/g in the crude extract (Figure 2) were quantified.

### 3.2. Croton Oil-Induced Inflammatory Parameters

**Figure 3:**
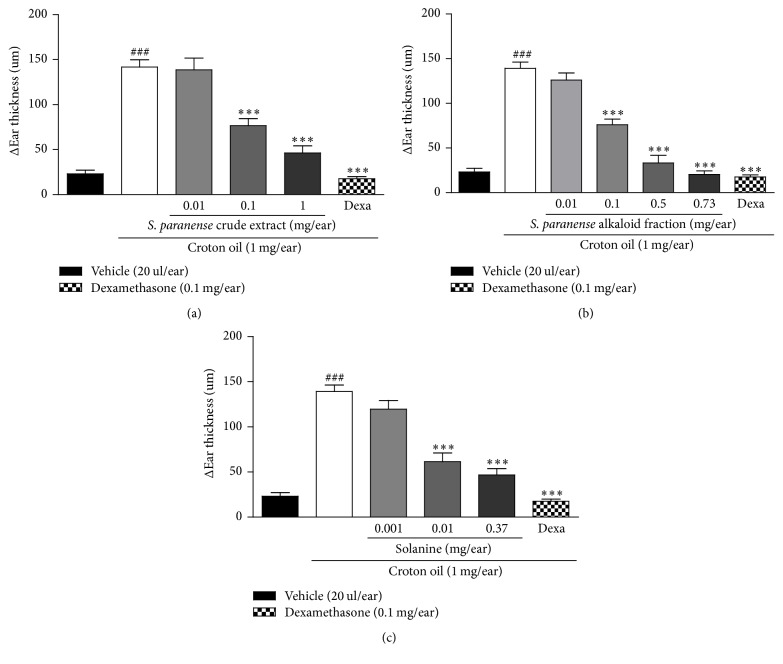
Effect of the* S. paranense* crude extract (a),* S. paranense* alkaloid fraction (b), solanine (c), and dexamethasone (Dexa) administered topically on croton oil-induced acute ear edema. Each bar represents the mean + SEM (*n* = 6); ^###^*P* < 0.001 when compared with the vehicle (acetone) group. ^*∗∗∗*^*P* < 0.001 when compared with the croton oil group (one-way ANOVA followed by post hoc Newman-Keuls test).

We assessed the anti-inflammatory activity of the crude extract, alkaloid fraction of* S. paranense, *and solanine in a croton oil-induced acute skin inflammation model. A single topical application of croton oil on the ear induced a marked increase in the ear thickness, with *E*_max_ of 142 ± 8 *μ*m when evaluated 6 h after the induction of inflammation process. On the other hand, the topical application of vehicle (acetone) alone did not significantly change the ear thickness (0.023 ± 0.04 *μ*m) (Figure 3(a)). This parameter is indicative of some processes that occur during skin inflammation, including increased vascular permeability, edema, and swelling within the dermis [13].

The crude extract (0.01–1 mg/ear), alkaloid fraction (0.01–0.73 mg/ear), and solanine (0.001–0.37 mg/ear) topically applied inhibited the croton oil-induced ear edema in a dose-dependent manner, with an ID_50_ value of 0.06 (0.03–0.12), 0.08 (0.06–0.11), and 0.004 (0.002–0.009) mg/ear and a maximum inhibition of 81 ± 7% (at 1 mg/ear), 98 ± 1% (0.73 mg/ear), and 80 ± 6% (0.37 mg/ear), respectively (Figures 3(a), 3(b), and 3(c)). Dexamethasone (positive control) inhibited the croton oil-induced ear edema with maximal inhibition of 100% (Figure 3).

**Figure 4:**
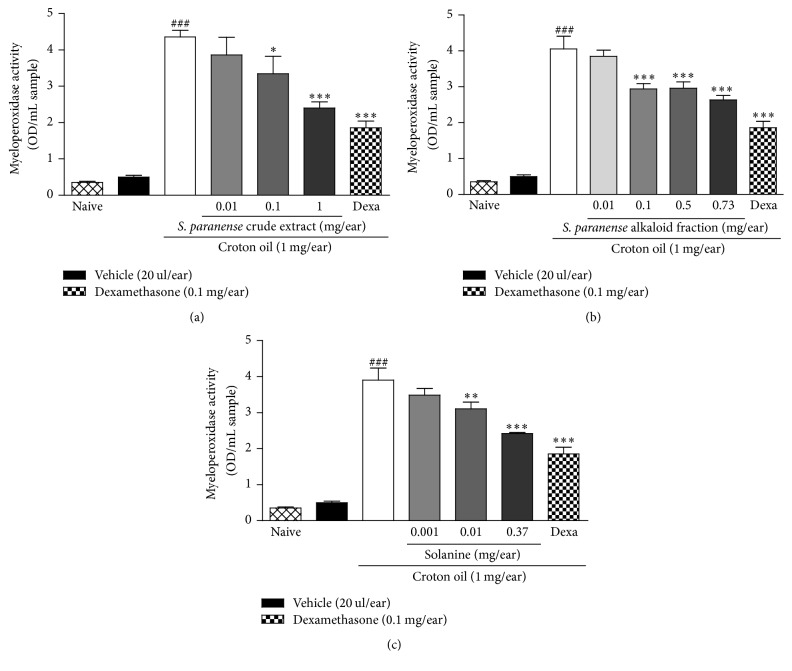
Effect of the* S. paranense* crude extract (a),* S. paranense* alkaloid fraction (b), solanine (c), and dexamethasone (Dexa) administered topically on MPO enzyme activity (OD/mL sample). Each bar represents the mean + SEM (*n* = 6); ^###^*P* < 0.001 when compared with the vehicle (acetone) and naïve groups. ^*∗*^*P* < 0.05, ^*∗∗*^*P* < 0.01, and ^*∗∗∗*^*P* < 0.001 when compared with the croton oil group (one-way ANOVA followed by post hoc Newman-Keuls test).

MPO is a biochemical marker of polymorphonuclear leukocytes and its activity is directly related to the amount of neutrophil infiltration, which is indicative of an inflammatory process. Croton oil caused an increase in the MPO activity when compared with the naïve group and the topical application of the crude extract, alkaloid fraction, and solanine was able to inhibit the croton oil-induced increase of MPO enzyme activity with an ID_50_ value of 0.04 (0.007–0.23), 0.05 (0.02–0.11), and 0.012 (0.004–0.036) mg/ear and a maximum inhibition of 51 ± 4% (1 mg/ear), 40 ± 3% (0.73 mg/ear), and 46 ± 1% (0.37 mg/ear), respectively (Figures 4(a), 4(b), and 4(c)) while dexamethasone reduced MPO activity in 65 ± 5% (Figure 4).

### 3.3. Histological Assessment of Ear Tissue

**Figure 5:**
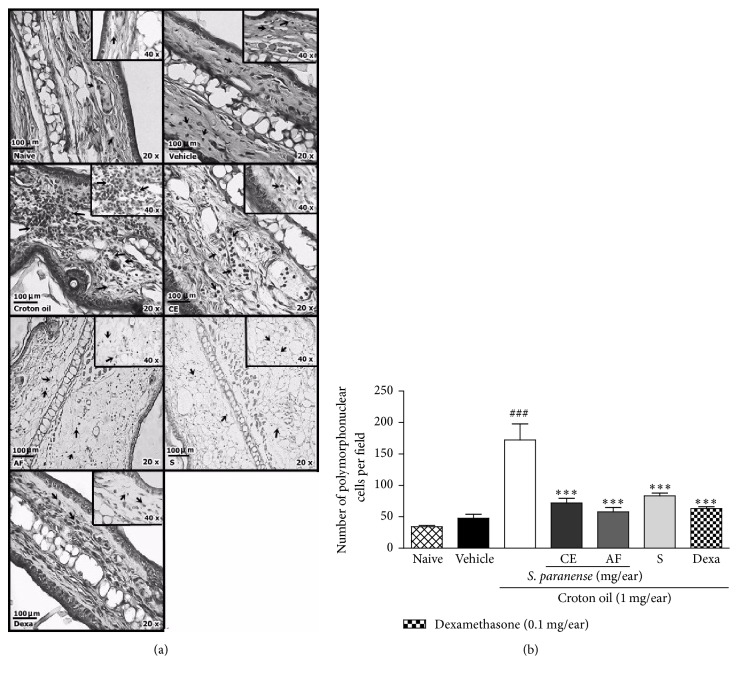
Effect of the* S. paranense* crude extract (CE; 1 mg/ear),* S. paranense* alkaloid fraction (AF; 0.73 mg/ear), solanine (S; 0.37 mg/ear), and dexamethasone (Dexa) (0.1 mg/ear) on histological changes in the ears croton oil-treated mice. Representative pictures of histological sections from mice ears stained with hematoxylin-eosin (20 and 40x increments; scale 100 *μ*m) (a) and counting polymorphonuclear cells per field (b) on acute skin inflammation model. The arrows (a) indicate polymorphonuclear leukocytes infiltration on the dermis. Each bar (b) represent the mean + SEM (*n* = 6); ^###^*P* < 0.001 when compared with the vehicle (acetone) and naïve groups. ^*∗∗*^*P* < 0.01 and ^*∗∗∗*^*P* < 0.001 when compared with the croton oil group (one-way ANOVA followed by post hoc Newman-Keuls test).

Once we observed the development of an inflammatory process after croton oil application, we investigated histological changes in the ear tissue at 6 h after the application of croton oil or croton oil plus treatments. Histological sections of the mice ears submitted to unique topical application of croton oil (Figure 5(a)) revealed the presence edema characterized by an intense increase of the ear thickness, especially at the dermis and expressive polymorphonuclear leukocytes migration, especially on croton oil group (172 ± 22 polymorphonuclear cells per field; Figure 5(b)), when compared with the naïve (34 ± 3 polymorphonuclear cells per field) or vehicle (48 ± 6 polymorphonuclear cells per field) group. The topical application of extract (1 mg/ear), alkaloid fraction (0.73 mg/ear), solanine (0.37 mg/ear), and dexamethasone (0.1 mg/ear) decreased the edema and inflammatory cells infiltration (72 ± 7, 58 ± 7, 83 ± 5, and 63 ± 3 polymorphonuclear cells per field, resp.) in comparison to croton oil group (Figures 5(a) and 5(b)).

## 4. Discussion

The scientific and empirical literature relates that several species of genus* Solanum* present skin antiedematogenic and anti-inflammatory activities [4, 14, 15]. Here, we show, for the first time, the anti-inflammatory activity of the solanine, crude extract, and alkaloid fraction of* S. paranense*. Solanine, alkaloid fraction, and crude extract of* S. paranense* were capable of reducing the ear edema and the inflammatory cell infiltration, demonstrated by MPO activity and confirmed by histological procedure, in a topical dermatitis model induced by croton oil application, indicating that the antiedematogenic activity is associated with decrease of inflammatory cells infiltration. Similar results were found by another work from our research group [4], which demonstrated that the extract of the genus* Solanum* leaves, species* Solanum corymbiflorum*, presents skin anti-inflammatory activity in a dermatitis model induced by croton oil. Moreover, Da Costa et al. [16] also showed that the hydroethanol fraction of the genus* Solanum* leaves, species* Solanum lycocarpum*, presents antiedematogenic activity in a carrageenan-induced paw edema model.

We demonstrated which* S. Paranense* extract has important polyphenols already found in other extracts of the genus* Solanum* and other genuses with described anti-inflammatory activity [17, 18]. Among the compounds with an anti-inflammatory activity, we highlight the rutin (21.85 mg/g) and rosmarinic acid (9.43). Rutin is a constituent found in large quantities in the* Viola tricolor* flowers which present anti-inflammatory effect in a burn model [17]. Rosmarinic acid showed an anti-inflammatory effect on ear edema and other models of inflammation through inhibition of adhesion molecule, chemokine, and eicosanoid synthesis and by its antioxidant properties [18, 19]. Zadra et al. [10] showed which rosmarinic and chlorogenic acid found in the* Solanum guaraniticum* extract are related to antioxidant activity; it is known that vegetal extracts with these effects have been established as a therapeutic approach for treating inflammation [20]. The chlorogenic and gallic acid found in the* S. paranense* extract probably contributed to topical anti-inflammatory activity, since another work from our research group [4] also found the same phenolic compounds in* S. corymbiflorum* extract which presented antiedematogenic and anti-inflammatory activities.

Research has shown that, besides polyphenols and flavonoids, alkaloids are often found in* Solanum* genus plants. Specifically, solanine was found in leaves of several species of this genus as* S. alandiae*,* S. phureja* spp., and* S. sparsipilum*, among others [21]. Moreover, the alkaloids possibly were also responsible for the anti-inflammatory activity of species of the* Solanum* genus [4, 22]. This way, solanine almost certainly is related to a topical anti-inflammatory activity of the* S. paranense* alkaloid fraction and also may act synergistically with other compounds as rosmarinic acid and rutin present in the* S. paranense *crude extract.

Research by Kenny et al. [23] showed that* Solanum tuberosum* peel extract, glycoalkaloid, such as solanine, and* S. tuberosum* peel extracts enriched in glycoalkaloids have anti-inflammatory activity in vitro. According to these authors, the aglycone unit of glycoalkaloids is essential for this effect because these nitrogen compounds are analogs of steroids saponins such as diosgenin, a molecule with proven anti-inflammatory activity.

## 5. Conclusion

In this research we confirmed for the first time that the extract and alkaloid fraction of* S. paranense* leaves possess antiedematogenic and anti-inflammatory activities in a croton oil-induced topical dermatitis model. These findings may be related with the solanine found in high quantity in the alkaloid fraction and also to a considerable quantity of phenolic compounds as rosmarinic acid and rutin present in the* S. paranense* extract.
